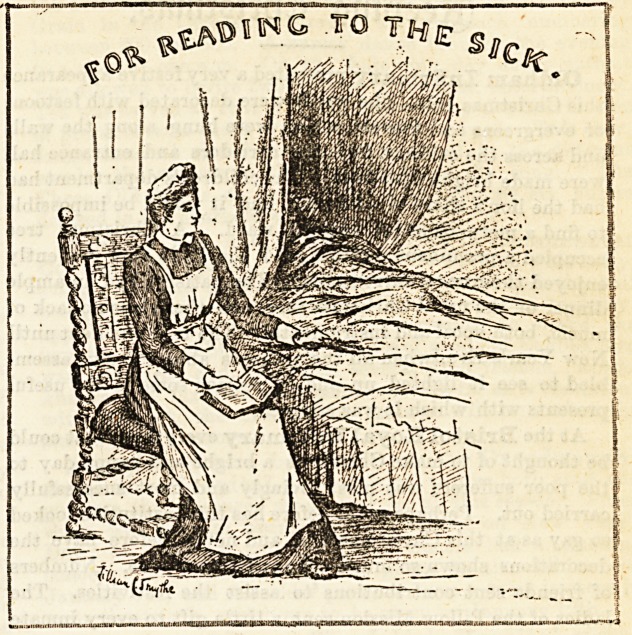# Extra Supplement—The Nursing Mirror

**Published:** 1891-01-10

**Authors:** 


Being the Extra Nursing Supplement of "The Hospital" Newspaper.

All Contributions for this Supplement should be addressed to the Editor, The Hospital, 140, Strand, London, W.C., and should have the word "Nursing" plainly written in left-hand top corner of the envelope.

### En Passant.

**Biblewomen and Nurses.**—The work of the London Bible and Domestic Female Mission, which was founded by Mrs. Ranyard in 1857, is still increasing. In London alone some 74 districts have been worked by 71 nurses, and there are seven nurses in training. Of course, the training is somewhat limited, the nurses being chiefly used as forerunners of mission-women. A full report and balance-sheet of last year's work has just been issued; it appears that the funds are rather low. The central office is at 2, Adelphi Terrace, Strand.

**The Hatchet Buried.**—There is peace at the Suffolk General Hospital, and everyone is complimenting every-one else, and everyone is swearing that the food is excellent and the hospital the best managed institution on earth. The moral of this is that no attention must be paid to complaints made by those in disagreement with the hospital officials. "Sarah Drury," the six months' pro., is quite forgotten, and relegated once more to those unknown regions from which she was dragged only to serve a cause. Let us hope there will be no more unseemly squabbling at Bury St. Edmunds.

**Mary Adelaide Letter.**—Miss Louisa Twining writes the nineteenth quarterly letter to the Mary Adelaide nurses, and especially directs their attention to the care of epileptics. Miss Twining points out forcibly the sad fact that there is no provision for epileptics of the middle class. It is a fact which is brought home to the editor every week; only two days ago we received a letter from a correspondent telling a tale of a servant girl subject to epilepsy, who fell in the fire and was terribly burnt. She is so disfigured by this accident she cannot find another situation, and she is absolutely destitute. Is there no philanthropist in England who will at once take up this subject, and see that some provision is made for these most wretched cases? Miss Twining, in her letter, describes the Bielefeld colony for epileptics, and the various homes in the United States. It is a disgrace that England should be behind other countries in this respect, and surely if the truth could be impressed on some rich man or woman they would put apart some of their wealth to supply this crying need of an institution for epileptics.

**Short Items.**—Lady Maud Wolmer is Hon. Secretary to a newly-formed branch of the Rural Nursing Association for Hampshire.—The general meeting of the Midwives' Institute and Trained Nurses' Club will be held on January 30th at six p.m.—A memorial is to be raised to the late Sister Harriet Mary, of the All Saints Sisterhood, who was for many years in charge of the Home for Incurables in Mortimer Street.—The Medical Secretary writes in the report of the Coldstream Cottage Hospital: "I have great pleasure in stating my satisfaction with the nursing, both in the hospital and district."—"The Patients' Record" is a small book compiled by an American nurse; it is a case book containing ruled spaces for all the nurses' observations, and also slips for the doctor's orders.—Mrs. Hewer commences a series of papers on "Chronic Cases" in this month's "Nursing Notes."—Miss Jones and several nurses suffered by the late robbery at Guy's; the thief found the door of the home opened, and walked in and carried off seven watches and several other articles of value.—Marylebone Infirmary held a New Year's Day reception which was largely attended by the friends of the nurses.

**The Nurses' Co-operation.**—Any private nurses interested in the combination of nurses for their own benefit—a scheme discussed in these pages last year—can hear how practical steps have been taken for its fulfilment by calling at 8, New Cavendish Street, Portland Place, W., between the hours of ten a.m. and four p.m.

**Personal News.**—Miss Bodger, Matron of the Hull General Infirmary, is leaving to be married.—Miss Abbott, Lady Superintendent of the Brompton Consumption Hospital, will shortly be married to one of the late house physicians.—Miss Stewart, the Matron of St. Bartholomew's, has been ill with diphtheria, but her many friends will hear with pleasure that she is recovering. A large number of Bart.'s nurses are on the sick list.

**Lewis's Nursing Charts.**—Amongst the many charts sent to us, few have pleased us so much as Lewis's Nursing Charts. They are very neat and concise, and sure to prove useful to nurses. Each chart is for 24 hours, and a case (price 1s.) to hold the charts is supplied by the makers, Messrs. H. K. Lewis, 136, Gower Street, W.C. We would suggest that these daily charts be used in acute cases in conjunction with the ordinary weekly temperature chart; the latter has the advantage of showing at a glance the rise and fall of the temperature curve, which is more difficult to estimate if the eye be carried from one daily chart to another. Lewis's nursing charts are sent free by post for 2s. for 50, or 3s. 6d. a hundred.

**A Nurse in Trouble.**—Nurse E. Owen, of the Birmingham City Asylum, has been found guilty of the manslaughter of Annie Lane, an epileptic patient. The deceased, who was a cripple, was taken from her bed and put in the bath, which contained three inches of water, scalding hot. The patient screamed with pain, and was immediately lifted out; but her body was seriously scalded, and she died the same afternoon. The coroner asked if the bath was used simply to save the delicate hands of the delicate nurses. Dr. Whitcombe, the asylum superintendent, said the water should have been tested by a thermometer before the patient was immersed. When his attention was called to the patient her scalds had been dressed, but she was suffering greatly from shock. He should say the temperature was at least 120 degrees.

**Bluebeard at Berry Wood.**—Dr. and Mrs. Greene, of the Berry Wood Asylum, gave their friends and patients a pleasant treat the other night, by the representation of an operatic medley, entitled "Bluebeard." Dr. Greene wrote the libretto, Mrs. Greene arranged and partly composed the music, Dr. Harding painted the scenery, and the dresses were arranged by Miss Evans. The head attendant led the band, and the programmes were printed by the asylum press. Of course the entertainment was most interesting, not only for its local flavour, but because of the real excellence of the performance and the spirit which animated all concerned. The opera was given on two nights, about 300 patients being present each time, also a large number of friends. The enterprise of the people at Berry Wood is worthy of wider imitation. It is impossible in the short space at our command to comment on the merits of individual actors, or to quote from the amusing verses of Dr. Greene, but we can say, most heartily, that we are sure these entertainments are most beneficial to the patients.

### Lectures on Surgical Ward Work and Nursing.

By Alexander Miles, M.B. (Edin.), C.M., F.R.C.S.E.

### Lecture IX.—THE DRESSING OF WOUNDS.

Washing the Wound.—A very common mistake made by students and nurses in washing a wound, is to clean up all the skin around the actual wound first, and then with the same swab, to rub over the granulations. In other words, they carefully gather up all the refuse lying around, and deposit it on the actively-absorbing part, the granulation tissue. Of course, the proper method is to clean the wound thoroughly at the very first, and having done so, never again to touch it. Then remove all the *debris* from the edges, and proceed with your dressing.

Protective.—The wound having been thoroughly cleaned, the application of the fresh dressing is to be proceeded with. As a rule, some form of protective is placed next the wound, and that most commonly used is Lister's oiled silk. As we have already seen, this has the great disadvantage of not being antiseptic, and on this account many surgeons have given up using it. As sold, it is between folds of tissue paper, and it should not be removed from these till just before it is required. Only cut as much as you require, and put it at once into antiseptic lotion, wash it thoroughly, and apply it directly from the lotion to the wound.

When oiled silk protective is not used, small pads of *unprepared or plain gauze* take its place. These have been kept for a fortnight or more in 1·20 carbolic, and so rendered thoroughly antiseptic. You should wash the carbolic out of the gauze by dipping it in your lotion, as the acid is apt to irritate the skin. These pads of gauze, in addition to being antiseptic, have the advantage of being absorbent of discharge, while they do not stick into the wound like wool.

Antiseptic Powder.—Next to the protective (sometimes under it) is dusted some antiseptic powder, iodoform, or boracic acid.

Deep Dressing.—Outside this is put what is known as the "deep-dressing." When unprepared gauze is used in place of protective no further deep dressing is necessary, but with oiled silk it is customary to apply some moist, absorbent, material just outside of it. The deep dressing may either be allowed to dry, or kept moist by a layer of gutta percha tissue over it. One of the most useful deep dressings is a square of boracic lint, 4-ply thick, wrung out of the lotion used for the dressing. Boracic lint so prepared will be found to absorb a large quantity of discharge. Outside the deep dressing another dusting of powder may be put, especially should the discharge smell.

The Wool.—This has already been so far prepared in arranging the dressing tray. It is there in large thick squares, but it must not be applied so to the wound. The amount applied will be proportionate to the size of the wound, and the amount of discharge expected. On the one hand you must guard against extravagance in the use of this rather expensive material, whilst on the other you must equally avoid a niggardly and false economy which saves wool at the expense of antiseptic efficiency. Rather use too much than too little wool. There are one or two points to which you must attend in applying the wool at a dressing. Never lift a piece of wool from the tray or box and put it straight on to a wound. Always split it up so as to get a fresh surface. The object in thus splitting up the wool is three-fold: (1) in order that you may have a fresh surface, on which no dust or germs may have landed to put next the wound; (2) that the wool may lie in more accurate apposition with the surface, so that it will not slip, and that air may not enter; and (3) that being loose and porous, the discharge will easily soak up into the wool, and not cake and form a hard impermeable layer next the wound.

In cases where a large amount of discharge is expected it will be well to use wood wool, taking the same precautions as to securing a fresh surface. It is more absorbent than ordinary corrosive wool, and this properly more than compensates for the inconvenience caused by the excess of dust it gives off. This, however, can be obviated by having it made up in sheets covered over with fine gauze.

Bandages.—Over the wool should be put some form of antiseptic gauze bandage to hold the dressing in position, either the gauze charged with the double cyanide of mercury and zinc, lately introduced by Sir Joseph Lister; or the older carbolised gauze of the same surgeon. Attend to the following points in connection with this bandage. You remember that carbolic acid is a volatile substance, and as the bandage you are going to hand up to the surgeon has doubtless lain in the tray for a day or two at least, the outer layers will probably have lost their antiseptic, and also will have had dust landing on them. This being so you should always tear off the first foot or foot and a-half of a gauze bandage before handing it to the doctor. Always select an appropriate width of bandage, and be careful to unrol a few inches of it before handing it up. This enables the surgeon to get started with the bandage at once, a thing which is not easy when a firm roll is given him and he has only one hand available for applying it, as is very often the case. As a rule, a domette bandage, charged with sal-alembroth, a non-volatile antiseptic, is applied over all to secure the dressing and to support the whole limb.

Safety Pins.—The bandage is fixed by means of safety pins, and you should always have a good supply ready, because it is often necessary to fix the bandage at several places, especially about the head, chest, and pelvis.

Few people insert a safety pin properly. The right way is to have the pin run in the long axis of the bandage. If you fix it in across the bandage the tension twists the pin round, and the last turn gets quite loose, and the other turns soon follow. The dressing is now finished, and it is your duty to make the patient comfortable again, and remove the dirty dressings, etc.

To Make the Patient Comfortable.—First remove the macintosh and dipped towel, and be careful in doing so to gather up the four corners, and then the intervening edges, to avoid soiling the sheets. Next quickly cover up your patient, and make him as comfortable as possible. See that the bed-clothes don't press on the injured limb. If so put in a "cage" to prevent this. Should the patient complain of being cold you may put a hot bottle beside him, taking care that it is not so hot, or so near the limb as to do damage. Never put a hot bottle beside an unconscious patient, a very old person, or one who has had a very severe injury to his limbs, without orders from the house-surgeon or staff-nurse, as it is liable to do harm.

Disposal of Dirty Dressings.—What is to be done with the dirty dressings which have been taken off? The domette bandage should be *at once* removed from the ward, and put into a basin of carbolic. It must on no account, however clean it may appear to be, be rolled up and put into the dressing-tray or used for another patient. If unstained, it may be used again for the same patient, but in wounds which it is important to keep aseptic—and there are few indeed which it is not—it is better never to use a bandage twice without having it thoroughly washed between times. Everything else should be at once burned, if this be possible. If not, remove it from the ward to a place where it can contaminate nothing, as soon as it comes off. On no account collect all the old dressings of the day in the ward, and remove them together at night. The carbolised towel should also be washed before being again used; and the macintosh must be thoroughly dried before being folded and laid aside. The dressing-tray should be tidied, the wool-boxes closed, and the whole covered over with a clean towel till it is again required. In some hospitals it is the duty of the nurse to mark on the chart at the proper place the word "Dressed," and any remarks necessary; and when it is, she should do it *at the time*, otherwise it is apt to be forgotten and to lead to mistakes.

*(To be continued.)*

### Queen Victoria Jubilee Institute for Nurses.

HER MAJESTY has been graciously pleased to approve of the following names being entered on the Roll of Queen's Nurses, for nursing the sick poor in their own homes :—

GENERAL INSPECTOR OF NURSES—Rosalind Paget.

SUPERINTENDENTS—

Emily Mansel Mansel, Bloomsbury.

Pauline Peter, Edinburgh.

Mary Robin, Kilmarnock.

Jessie Mackay, Dundee.

Mary Lucy Eliza Dunn, Dublin.

Jane Elizabeth Heath, Cardiff.

Emmiline Stains, Liverpool.

Harriet Isabella Moore, Liverpool.

Mary Howarth, Liverpool.

Sara Wilson, Liverpool.

Agnes Wells, Haggerston.

Amy Hughes, Chelsea.

Agnes Eliza Spring, Hampstead.

Ada Booth, Kensington.

Lucy Osburn, Newington.

Florence Edith Thomas, Bolton.

Arabella Elizabeth Stone, Westminster.

Catherine Eliza Barff, Manchester.

Esther Amelia Hind, Manchester.

Jerrie Blower, Manchester.

Elizabeth Ann Holloway, Worcester.

Helen Ryan, Camberwell.

Total, 22.

NURSES—

Grace Gordon, Bloomsbury.

Maggie Wilson Smith, Edinburgh.

Jessie Mackay, Edinburgh.

Mary Caris, Edinburgh.

Mary Mackechnie, Edinburgh.

Alice Mary Sanders, Kensington.

Florence Matilda Rudyard, Kensington.

Mary Katherine Haines, Newington.

Lucy Catherine Murray, Newington.

Mary Parry, Bolton.

Annie Marriott, Manchester.

Bessie Kelly, Manchester,

Annie Simpson, Manchester.

Sarah Annie Mills, Manchester.

Edith Annie Lawson, Manchester.

Eliza Ratcliffe, Manchester.

Clara Boddington, Manchester.

Ann Jane Evans, Manchester.

Mary Elizabeth Pickering, Manchester.

Minnie Irvine Scott, Manchester.

Mary Louisa Holyoake, Manchester.

Harriett Ella Steele Davies, Manchester.

Ellen Healy, Manchester.

Sarah Frances Lake, Manchester.

Mary Ellen Bennett, Manchester.

Mary Bird, Worcester.

Mabel Jane Hamilton Rowland, Worcester.

Caroline Lucy Allerton, Worcester.

Jane Beveridge Wilson, Worcester.

Marian Matthews, Worcester.

Grace Harriet Tyson Rees, Worcester.

Mary Jordon, Worcester.

Mary Ravenscroft, Worcester.

Mary Ann Walters, Worcester.

Selina Leech, Worcester.

Annie Maria Deville, Worcester.

Alice Mary Wallis Spooner, Camberwell.

Elise Dehn, Little Haven.

Total, 71.

St. Katharine's Royal Hospital, Regent's Park, N.W., January 1st, 1891.

### Appointments.

[It is requested that successful candidates will send a copy of their applications and testimonials, with date of election, to THE EDITOR, The Lodge, Porchester Square, W.]

EAST LONDON HOSPITAL FOR CHILDREN.—Miss Tolhurst has been appointed out-patient sister; she trained at Gt. Ormond Street Hospital for Children.

CIRENCESTER COTTAGE HOSPITAL.—We hear with pleasure that Miss Alice Mary Glover has been appointed Matron of this hospital.

WORTHING DISTRICT SOCIETY.—Miss Baskett, of the Metropolitan and National Association, has been appointed district nurse at Worthing.

### Death in Our Ranks.

NURSE ALICE EMMA PHILLIPS (age 20) of typhoid fever. She only resigned her post at Paddington Green Children's Hospital on December 22nd; upon reaching home felt ill, took to her bed, and died in the Norwood Cottage Hospital, January 1st. Nurse Alice had acted as probationer formerly at Norwood for nearly two years.

### THE OLD YEAR.

WE all like the idea of anything new, it is an enticing thought, especially to the young, and when we think of the New Year it is doubly so to everybody. For there are such possibilities in the future to a hopeful disposition. What a number of things might happen in the 365 days which stretch before us, for our happiness! It might be also for sorrow, but we cast away the latter thought and keep to the brighter side of the picture.

Here, now, we have a fresh start in life, and we determine we will turn over a new leaf with the New Year and try to be wiser, steadier, kinder, better in every way than in the past. Or we are very sad and disconsolate and we hope that God will remove the cause of our sorrow; or we are sick and suffering, and we trust that He will abate or even cure our disease. At any rate, we know that He will give us a happy issue out of all our afflictions if we ask with all our hearts.

It is good for us in every way, both for body and mind, to take a hopeful view of the future, but we ought also to look back a little at the year which has "fled beyond recall," and at the mercies and blessings which we received in it, and which we have left behind. Surely we must feel a tender regret when we ponder on the fact. Shall we ever have another twelve months quite so pleasant or prosperous? And those who have been blessed with a renewed health and vigour must perforce pour out their hearts in gratitude and thanksgiving. While others have fallen victims to numberless accidents, or sudden death from various causes, we have been spared and are alive at this day. "The dead praise not Thee, O Lord, neither all they that go down into silence. but we will praise the Lord from this time forth for evermore. Praise the Lord."

We will begin the year, then, with regret for our backslidings, and joy for our mercies in the past, mingled with good resolutions for the future.

And we will not be selfish in our griefs and joys, our hearts shall go out in sympathy with all. We will thankfully praise the Lord for His countless gifts to all mankind. We will ask Him not only to defend ourselves but our country and to give it peace and plenty in the coming year. We will beseech Him to restrain the growth of vice and to grant that our future may be spent in His service and in striving to gain that crown of righteousness which will be given to those who conquer in the fight.

O make but trial of His love,
Experience will decide
How blest are they and only they
Who in His truth confide.

### Keeping Christmas.

**Oldham Infirmary** presented a very festive appearance this Christmas tide. The wards were decorated with festoons of evergreens and flowers, which were hung along the walls and across the ceilings, and the corridors and entrance hall were made bright with flags. The children's department had had the lion's share of attention, and it would be impossible to find a more cheerful hospital ward. A Christmas tree occupied a prominent position, and the little ones evidently enjoyed their novel surroundings. The patients had an ample dinner of the usual Christmas fare, and there was no lack of music, both vocal and instrumental. The tree was kept until New Year's Eve, when all the patients able to move assembled to see it lighted up and to receive some of the useful presents with which it was adorned.

At the **Bristol Royal Infirmary** everything that could be thought of to make Christmas a bright and happy day to the poor sufferers was most lovingly and most successfully carried out. Perhaps never before has this institution looked so gay as at this Christmas-tide, and never before have the decorations shown so much originality and taste. Numbers of friends sent contributions to assist the festivities. The ladies of the Pillow Mission sent a little gift to every inmate of the hospital, and a large number of presents were sent for distribution in the children's ward, where, in consequence, great happiness prevailed. The day passed enjoyably for all; and next week the medical and nursing staff will give a dramatic entertainment, so the patients have still something to look forward to.

The little inmates of the **Birmingham Children's Hospital** had their festivities on Christmas Eve. An old friend of the children, the Rev. E.P. Gounor, acted as Father Christmas, and went from bedside to bedside, distributing gifts and cheering words, recognised in spite of his disguise by the majority of the children. So bountifully was he supplied that the children rec eived not one, but two toys, besides the chocolates so kindly sent by Messrs. Cadbury. Nor was this all, for after the toyman had gone his rounds the trees were lighted. This year there were no less than seven beautifully-dressed trees. They are kept up until New Year's Eve, when they are taken down and stripped, but the toys are not at once distributed, but are carefully put aside, and each child receives one as it leaves the hospital.

In the **Dartmouth Cottage Hospital** Christmas was spent in a very bright and cheery manner. On Christmas Eve the Matron kindly invited the wives and children of the patients to tea, which was served in the men's ward. The ward had been beautifully decorated by the help of Miss Edith Tew, who also assisted the Matron to amuse the little folks. A large Christmas tree, provided by the Matron, was placed in the centre of the ward, and off this both children and patients received gifts either of toys or something useful. On Christmas day a service was held in the ward by the chaplain, accompanied by his choir boys, who gave much pleasure to the patients by singing carols. The Matron was well rewarded for the pains she took to make her patients happy by one of them saying he "had been as happy as if he had been at home."

At the **Bristol General Hospital** the patients had their comfort well looked after. Each of the wards had its own particular mode of ornamentation, and considerable taste was shown in the selection of some of the mottoes. The cheerful faces of the patients showed how much they appreciated the efforts put forth to increase their happiness. In the children's ward there was a large Christmas tree and a very pretty representation of "Little Red Riding Hood."

A happy Christmas was spent by the patients in **Rotherham Hospital and Dispensary**. The Board according to their custom, provided a liberal dinner of Christmas fare, and kind friends sent presents of dessert and tobacco. The patients were entertained with an excellent concert, which both they and their visitors thoroughly enjoyed. Everybody in the hospital received some little gift to remind them of this happy day.

Christmas at **Dorchester County Hospital** was a happy day for all connected with the institution. The nurses had undertaken the decorations, and the result was most satisfactory. Appropriate texts adorned the walls, and the usual evergreens were brightened by Chirese lanterns and fairy lamps. In the morning each of the patients received a present, and then those who were well enough attended the service in the chapel. After a plentiful repast of roast beef and plum pudding, all who could be moved assembled in one of the female wards, where they were entertained by the Matron and some friends with songs and recitations. A concert and a Christmas tree are to be given early in January.

At the **Brompton Hospital** much has been done to render the Christmas season bright and enjoyable for the patients. The festivities of last week, have now been supplemented by an entertainment, for which a lofty Christmas tree, hung with pretty trifles, had been provided by the Misses Heddy. Tables were laid out with a large number of parcels containing useful gifts for every inmate of the hospital, besides which were a quantity of toys from the *Truth* collection.

The usual Christmas festivities were observed in the **Birmingham City Hospital.** The wards, both at the Lodge Road Central Hospital and at the Western Road and Landsdown Street branch hospitals, were very gaily decorated. Evergreens, appropriate mottoes, and designs adorned the walls. All of these were made by the members of the staff, who deserve great credit for the artistic taste which they displayed, and also for the amount of trouble and labour they spent in promoting the happiness of their patients. Fourteen Christmas trees were loaded with toys, and the children in particular were delighted with them. After a good dinner, the convalescent patients thoroughly enjoyed the amusements provided for them, and all spent a happy and enjoyable day.

At **Darlington Hospital** the patients were entertained by a brightly-written dialogue between a nurse of a hundred years ago and a nurse of to-day. The two nurses were dressed in costumes, typical of the times they represented, and as their dialogue was exceedingly clever and amusing, the patients enjoyed some very hearty laughs.

At the **York County Hospital,** Christmas was kept in a very hearty manner. The wards were plentifully and artistically decorated, and looked exceptionally bright and pretty. The Christmas tree entertainment, which was most successful, was opened by the Dean, and was attended by a large number of visitors.

The friends of **Bradford Children's Hospital** made hearty efforts to make Christmas a day of unusual cheeriness to the forty-four little inmates at present detained beneath its roof. An abundant supply of toys and decorations was supplied by the sympathisers and helpers of the institution. The usual Christmas dinner to the patients and staff, was given by the mayor, who, with a party of friends, made a tour of the wards during the afternoon. Mrs. Charles Kesler made the day memorable by the donation of £200 for the endowment of a cot.

The inmates of the **Bristol Eye Hospital** were made as comfortable as possible, and the patients seemed heartily to appreciate the kindly care of the Matron. Their dining-room was made to look cheerful and pretty with appropriate texts hung on the walls, and shields of red cloth, with edgings of evergreens. The patients were regaled with a capital dinner of roast beef, turkey, plum pudding, mince pies, &c., after which beer, wine, and tobacco were provided. The men were presented with scarves and neckties and the women with shawls and mittens. In the evening musical selections were given, and everyone did their best to give the patients a happy Christmas.

At the **Lockhart Hospital, Lanark,** there were the usual festivities; a Christmas dinner for patients and handsome presents for the servants. A large party of children, former patients, and the friends of present patients came in the afternoon. The ward was decorated with evergreens and the tables prettily laid out. The ladies from the Lee assisted to wait on the children; afterwards the children amused themselves for four hours, and left laden with good things.

The **London Hospital** had various entertainments in the wards on different nights, but the chief Christmas festivity is the reception in the Queen, or children's, wards, which took place on December 30th. The wards were beautifully decorated, and the friends of the hospital mustered in great force, and were heartily welcomed by the Matron who was looking in much better health. There was a Punch and Judy show, and then a Christmas tree and distribution of toys. Sponge cakes and oranges concluded the day.

Shadwell looked its very worst with mud and fog on the 2nd, when the **East London Hospital for Children** held its festivities; but inside the hospital all was bright and cheerful. The decorations were very pretty, especially a peal of bells in the board-room. All the wards were open to visitors, and then at five o'clock a procession of nurses, carrying wee helpless patients came along the corridors, and all the little sick folk were gathered in one room, and amused by their old, old favourite, Mr. Punch.

The inmates of the **General Hospital, Nottingham,** were not forgotten by the kind friends who annually help them to forget their sufferings. Miss Wright distributed her customary gifts on Christmas Eve, and Dr. Wright sent his usual box of oranges. The Misses Hopkins sent some pretty little New Testaments, and a quantity of new sixpences for the children. Other friends sent materials for the decorations, and the Christmas Day fund supplied several pounds of tobacco and a liberal dinner of roast beef and plum-pudding. The patients were gratefully appreciative of all the kindness shown them.

At the **Metropolitan Hospital** an entertainment was given last Tuesday. The wards were prettily decorated with flags and fairy lights, and one of the staff in the garb of Father Christmas distributed gifts. A concert, in which the Defries family (ever good friends to the hospital) chiefly performed, was much appreciated by the patients, some of whom were present in their beds. There was also a cleverly-acted comic drama.

The wards of the **University College Hospital** presented a gay appearance in the festive attire they donned for Christmas. A tree heavily laden with toys was provided for the children out of a fund raised by the Sister Superior, amounting to nearly £100. This fund also secured for every patient and servant in the hospital a useful present of clothes adapted to their various wants. The principal entertainment was a Christmas tea in each ward, and a concert for convalescent inmates in the out-patients' ward; and for the amusement of those who could not leave their beds, glees were sung in the different wards. The 207 beds were all occupied, and a large number of friends visited the institution during the day.

Everyone connected with the **Poplar Hospital for Accidents** was untiring in the effort to mitigate the sufferings of the patients and make their lot easier to bear. Christmas Day began with carols, which were sung by the choir boys of a neighbouring church. Chickens, which the Matron had received as a present and kindly handed over to the patients, were a feature in the bill of fare, which included plum pudding and other Christmas fare. A huge tree had on it presents for everyone, of toys or of a useful nature, which were greatly appreciated by the recipients.

As a Christmas treat at the **Sussex County Hospital** all the usual restraints were withheld. The day began with carols, sung by a choir of nurses, who started at four a.m., and patrolled the wards. Not only were the men presented with pipes and tobacco, but cigarettes were given to the female patients. On December 30th an entertainment was given to the friends of the nurses. It was attended by many of the supporters of the hospital. The chief amusement of the evening was two musical sketches, given by Mr. Corney Grain in the Chichester ward, his audience numbering between 200 and 300. A nurses' dance brought the evening to a close at one a.m. On the following day every patient was allowed to invite two friends to a varied entertainment. Tea was served at four o'clock, followed by the distribution of presents from the tree in the children's ward. This over, songs, musical sketches, &c., were given in the different wards, the success of the evening being the song, "The Three Old Maids of Lee," which was sung by six nurses dressed in the characters.

Christmas Day was happily observed in **Cirencester Cottage Hospital.** The walls were covered with holly, evergreens, and mottoes, the children's ward and the hall being specially well trimmed. Miss Glover, the recently-appointed Matron, did everything possible to make the inmates comfortable. An abundance of good old English fare was provided, and, thanks to several kind friends, knick-knacks and luxuries were plentious. So far as is consistent with their lot, the unfortunate inmates will remember the season with pleasure.

Arrangements have been made at the **Dreadnought Seamen's Hospital,** at Greenwich, to give both staff and patients the pleasures we all look for at this season. First, there was a social evening for the nurses on New Year's Day; on January 2nd the petty officers had a smoking concert; a Christmas tree was given to the children on January 7th, and the older patients have a treat in store in some theatricals, which are to be given on January 12th.

The patients of **Queen Charlotte's Hospital,** spent a very happy Christmas Day, and were very pleased with the present each received, viz., a complete suit of baby clothes; any patient without a baby received a flannel petticoat. They enjoyed their dinner (turkey); they had cake for tea. Altogether they had a pleasant day, and were very grateful to their benefactors.

A serious mishap somewhat marred the enjoyment of the Christmas festivities at **Peterborough Infirmary.** The dispenser was acting as Father Christmas, and wearing a flowing beard of cotton wool. While he was distributing articles from a Christmas tree his beard caught fire, instantly enveloping his head in flames. His face, ears, and head were badly burnt, but it is hoped the injuries will not be of a permanent character. Panic among the patients was fortunately prevented.

At the **Children's Hospital, Nottingham,** Miss Morse never allows the little sufferers under her care to miss any of the little pleasures that may be associated in their minds with this season. The thirty children now in the hospital were made happy with presents of dolls, toys, and books sent by generous and thoughtful friends. After a tea, at which several former patients were present, a ship, cleverly built by one of the nurses, was carried round the wards, and the cargo distributed. The parents of the children were enabled to visit them throughout the day.

Over 120 past and present patients and friends of the **Princess Alice Hospital, Eastbourne,** gathered in one of the wards, the occasion being the annual distribution of gifts from a Christmas tree. Every patient received a present, and the gifts included toys for the little ones, and articles of clothing for the elders. The distribution took place in an empty ward in the new wing, which had not yet been furnished. The children indulged in several games.

At the **Royal Albert Hospital, Devonport,** a New Year's treat was held, the Matron, Miss Rimbault, and Sister Katherine, having dressed a very handsome tree. During the afternoon several of the ladies present gave items of vocal and instrumental music. During the Christmas week Miss Dunstan, the Matron, was the recipient of a very pleasing token of the respect in which she is held by the sisters, nurses, and probationers of the Hospital, who presented her with a splendid hand-painted photograph cabinet, and a ladies' mirror, with side glasses.

### LOVE.

"I wonder why it is that we are not all kinder than we are? How much the world needs it! How easily it is done! How instantaneously it acts! How infallibly it is remembered! How superabundantly it pays itself back! for there is no debtor in the world so honourable, so superbly honourable, as Love."—*Prof. Drummond.*

### The Brimsby Ghost.

*(Concluded from page lxxvi.)*

"Then you've seen it? —— " began Frank, ecstatically.

"No, we've not. But each morning, regularly, the book-marker Noll uses, in every volume he reads, is moved some pages farther on. To-night Noll will, as usual, note the page, and you'll see for yourself that marker moved to-morrow morning."

"Let us talk of something else," begged Aunt Sophy; "it makes me, and Nancy, too, so nervous."

Though we complied, we thought of nothing but the eerie Presence which had invited itself to Brimsby. My nerves are good, but I admit my night was sleepless as I pictured the ghostly reader below, in the quiet study. Morning found us all, before breakfast, regarding, with bated breath, the displaced marker.

"IT must have read twenty pages!" I cried, bending down to count. As I did so, I became aware of a faint, subtle perfume from the pages. If there be one sense I possess in all its perfection—sometimes to my sorrow—it is that of scent; I might have been distinguished even as an Indian—or a pointer. For a brief moment I was motionless, while my brain followed up the trail of the odour my nose had detected. Then my eyes rested on Nancy, slim and fair, as she indolently leant against Oliver's chair; Nancy, the *Mary*, in one sense, of the family, who sat with folded hands; whose aim in life was to make her young beauty still fairer with dainty apparel and exquisite perfume.

There was much said though little done that day, about the ghostly presence that had come to disturb the peace of the Rectory-house. I could see that Aunt Sophy and the rest were unhinged; they were totally unlike their ordinary equable selves; even Oliver was affected. They tried their best to edge off the subject, but it incessantly cropped up, and Frank was in a fizz.

"I'll sit up all to-night in your chair, Oliver," he declared.

"That you shan't, my dear!" said I promptly, "You're hiding a sore throat as it is, and sitting up in the cold would just send you over the precipice into an illness."

That was enough for Aunt Sophy. She has considerable respect for my medical knowledge, so I knew nobody would sit up that night—except one person whom I intended to be myself.

The lights were all out, and the fires had all died away, leaving an unearthly chill in the rambling old house. Wrapped up in my fur cloak, I shivered as I waited for the footsteps of the Presence, my room-door ajar.

It was past two before they came, softly pit-a-pat, past the door. My heart beat heavily. Presently I could hear the study door quietly opened, then shut. Now was my time! Feeling my way down the stair-case, I groped for the study door. Dare I turn the handle? Forcing myself, I did so.

There sat the Ghost; in her white hands Oliver's book, which by the dim light of a tiny silver lamp, she was carefully reading, out of wide, stony, dead-looking eyes.

Nancy was the mystery, and I had discovered it from the perfume (stephanotis) which she incessantly used. Of course, it was a case of sonnambulism, and not daring to disturb her, I stole noislessly away, determined that measures should be taken to cure the damsel.

Next morning the excitement was tremendous when the secret came out. Never were people more relieved than the other Trents; and the reaction culminated in, certainly, the most uproariously Merry Christmas Brimsby had ever known.

### Where to Go.

A performance in aid of the New Hospital for Women, 144, Euston Road, has been arranged to be given at St. George's Hall, Langham Place, on Saturday evening, January 17th, when the Cavendish Dramatic Society (employés of Messrs. Debenham and Freebody), will, by special request, repeat their recent successful performance of Godfrey's Comedy "The Parvenu."

The Moore and Burgess Minstrels are well worth a visit; they perform daily at 8 p.m., at St. James's Hall, and on Mondays, Wednesdays, and Saturdays, at 3 p.m.

The exhibition of Old Masters, at the Academy contains some lovely landscapes; in the water-colour room are a number of Turners and a few good De Witts, Fred Walker's "Marlow Ferry," and other popular paintings are also on view.

### Presentations.

The nurses of Great Ormond Street presented their late Lady Superintendent, Miss K. Philippa Hicks with a handsome tea-set in silver; they also presented the late House-Surgeon, Mr. Brooke, with a Gladstone bag; and the late House-Physician, Dr. Bayes, with a set of apostle spoons and a copy of George Eliot's works. The nurses must have been extremely fond of those late in authority over them, and must certainly be a very generous lot of women.

The Lady Superintendent of the Nurses' Institute, Weston-super-Mare, was much pleased on New Year's morning with the presentation of a very handsome pollard oak writing cabinet, by her nurses, as a token of their love and esteem.

### Notes and Queries.

### Queries.

(25) *Washing Blankets.*—What is the best way to wash scarlet blankets or counterpanes?—*M. H.*

(26).—Could any reader kindly give any information as to where a home in the country could be found for a gentleman suffering from several complaints, and who is able to pay a reasonable sum weekly?—*A. G. Farrer.*

### Answers.

(23) *Nursing Institutions.*—The Sussex County Hospital at Brighton has a private institution connected with it, which has been a great success. Doubtless the Matron, Miss Scott, would give particulars.—*Bee.*

*Aron.*—There are no such things as brown paper blankets; a sheet of ordinary brown paper is often used by the poor for warmth when blankets are scarce.

### Amusements and Relaxation.

### SPECIAL NOTICE TO CORRESPONDENTS.

First quarterly word competition commenced January 3rd, 1891; ends March 28th, 1891.

Competitors can enter for *all* quarterly compentions, but no competitor can take more than *one first prize* or *two* prizes of any kind during the year.

Three prizes of 15s., 10s., 5s., will be given for the largest number of words derived from the words set for dissection.

N.B.—Word dissections must be sent in WEEKLY not later than the first post on Thursday to the *Prize Editor*, 140, Strand, W.C., arranged alphabetically, with correct total affixed.

The word for dissection for this, the SECOND week of the quarter, being "CARNIVAL."

| Names. | Jan. 3rd. | Totals. | Names. | Jan. 3rd. | Totals. |
|---|---|---|---|---|---|
| Jenny Wren | 53 | 748 | Checkmate | — | 76 |
| Tinie | — | 55 | Silver King | — | 163 |
| Agamemnon | 52 | 770 | S. Anthony | — | 76 |
| Patience | 53 | 762 | Quackah | — | 75 |
| Ecila | 54 | 7[illegible]2 | Reynard | — | — |
| Lightowlers | 55 | 739 | Sally | — | 27 |
| Rouge | — | 89 | Success | — | 61 |
| Wyamaris | 54 | 763 | Caledonia | — | 52 |
| Qu'appelle | 48 | 659 | Nurse Emma | — | 305 |
| Nosam | — | 570 | Hazel | — | 20 |
| Nurse Hilda | — | 44 | Pallas | — | 48 |
| Lady Betty | 49 | 699 | Puss | — | 15 |
| Grenelle | — | 43 | Shakespeare | 20 | 595 |
| Daisy | — | 324 | Melita | — | 576 |
| H. A. S. | — | 157 | Nora | — | 10 |
| A. B. C. | — | 66 | Elsie | — | 35 |
| Liz | — | 444 | Espèrance | — | 27 |

### Results of Fourth Quarterly Word Competition.

Wyamaris (Miss Whaley, Barnsley, Yorks) is awarded the *First Prize*, 15s., for second highest total of words; *Mr. H. Hull*, Weybridge, heads the list, but having already received the *first prize once* during the year, he is, according to rule, ineligible to take it again; we therefore award him the *Second Prize*, 10s. The *Third Prize*, 5s., is divided between Ecila (Miss E. A. Todd, Moulsoe, Newport Pagnell), and Patience (Miss Cal sé, Newbury), whose totals are equal.

## Figures and Tables

**Figure f1:**